# Preserved Capacity for Adaptations in Strength and Muscle Regulatory Factors in Elderly in Response to Resistance Exercise Training and Deconditioning

**DOI:** 10.3390/jcm9072188

**Published:** 2020-07-10

**Authors:** Andreas Mæchel Fritzen, Frank D. Thøgersen, Khaled Abdul Nasser Qadri, Thomas Krag, Marie-Louise Sveen, John Vissing, Tina D. Jeppesen

**Affiliations:** 1 Department of Neurology, Copenhagen Neuromuscular Center, Rigshospitalet, DK-2100 Copenhagen, Denmark; frank.thogersen@gmail.com (F.D.T.); khaled_qadri@hotmail.com (K.A.N.Q.); thomas.krag@regionh.dk (T.K.); mqsv@novonordisk.com (M.-L.S.); john.vissing@regionh.dk (J.V.); tina@dysgaard.dk (T.D.J.); 2 Molecular Physiology Group, Department of Nutrition, Exercise, and Sports, Faculty of Science, University of Copenhagen, DK-2100 Copenhagen, Denmark; 3 Novo Nordisk A/S, DK-2860 Søborg, Denmark

**Keywords:** resistance exercise training, muscle regulatory factors, sarcopenia, muscle strength, deconditioning, skeletal muscle, elderly, hypertrophy

## Abstract

Aging is related to an inevitable loss of muscle mass and strength. The mechanisms behind age-related loss of muscle tissue are not fully understood but may, among other things, be induced by age-related differences in myogenic regulatory factors. Resistance exercise training and deconditioning offers a model to investigate differences in myogenic regulatory factors that may be important for age-related loss of muscle mass and strength. Nine elderly (82 ± 7 years old) and nine young, healthy persons (22 ± 2 years old) participated in the study. Exercise consisted of six weeks of resistance training of the quadriceps muscle followed by eight weeks of deconditioning. Muscle biopsy samples before and after training and during the deconditioning period were analyzed for MyoD, myogenin, insulin-like growth-factor I receptor, activin receptor IIB, smad2, porin, and citrate synthase. Muscle strength improved with resistance training by 78% (95.0 ± 22.0 kg) in the elderly to a similar extent as in the young participants (83.5%; 178.2 ± 44.2 kg) and returned to baseline in both groups after eight weeks of deconditioning. No difference was seen in expression of muscle regulatory factors between elderly and young in response to exercise training and deconditioning. In conclusion, the capacity to gain muscle strength with resistance exercise training in elderly was not impaired, highlighting this as a potent tool to combat age-related loss of muscle function, possibly due to preserved regulation of myogenic factors in elderly compared with young muscle.

## 1. Introduction

Sarcopenia means loss of flesh. The term is used to describe the pathological age-related loss of muscle mass, function, and strength that inevitable occurs in humans [1]. From the age of 50 to 85, humans lose 50% of their muscle mass, which is mainly a result of loss of type II muscle fibers [2]. Age-related loss of muscle mass and strength is associated with increasing risk of falling and disability, and thus impairment of basic daily activities.

It is well established that resistance exercise training can counteract the age-related changes in contractile function, strength, hypertrophy, and morphology of aging skeletal muscle [3]. However, whether the potential to adapt to resistance exercise training is completely preserved in skeletal muscles of elderly has been debated. [3]. Although 6–10 weeks of exercise training increased skeletal muscle strength to a similar extent in young and elderly in some studies [4,5], others report greater improvements in young individuals [6,7]. The rate of decline in muscle strength with age is 2–5 times greater than declines in muscle size [8] and strength loss is highly associated with both mortality and physical disability, even when adjusting for sarcopenia, indicating that muscle mass loss may be secondary to the effects of strength loss [9]. It is thus of key interest to elucidate whether increased muscle strength after a period of resistance exercise training occurs to a similar or blunted extent in old compared to young muscle. Moreover, although an aged-associated loss of muscle mass or strength [10,11] appears improved with resistance training in elderly individuals [12,13,14,15,16], several studies find a blunted muscle hypertrophy response [17,18,19].

Mechanisms responsible for resistance exercise-induced muscle hypertrophy are numerous, but some of the key factors include MyoD, myogenin, and insulin-like growth factor-I (IGF-I) [20,21,22]. The myogenic regulatory factors (MRF) are transcription factors that promote and regulate the expression of muscle-specific genes, which are essential to the hypertrophic and regenerative response following resistance exercise [23,24,25,26]. As MyoD is highly involved in muscle adaptation to resistance exercise, this has been a key factor in studies investigating potential differences in age-related muscle loss [27,28,29]. Differences in MRFs between young and elderly could be crucial mechanisms behind differences in muscle mass and strength [22,23,30] and in the response to resistance training and deconditioning. Previous studies found elevated levels of MyoD, myogenin, and IGF-I-R mRNA in elderly both at rest [17,31,32,33,34] and in response to one bout of resistance exercise [11]. Moreover, 16 weeks of resistance training increased muscle myoD mRNA levels to a similar extent in young and elderly, whereas the training-induced increase in mRNA levels of myogenin was impaired in the elderly [17].

Proteins responsible for negative muscle mass regulation, e.g., actRIIB and smad2, could be upregulated in inactive, aged muscle and be attenuated in response to resistance exercise, resulting in decreased muscle mass. In support, the mRNA level of actRIIB was downregulated after 21 weeks of resistance exercise in elderly [35,36]. The regulation of these molecular pathways involved in negative muscle mass regulation in response to resistance training in young and old muscle is unknown.

Mitochondrial dysfunction is another suggested key contributor loss of muscle mass with age [30]. Resistance exercise training has been found to affect mitochondrial function [37] evidenced by improved mitochondrial respiration and complex protein content after 12 weeks of resistance exercise training in young men [38]. However, whether resistance exercise training induces markers of mitochondrial content in elderly to a similar extent seems not clear.

In the present study, we therefore investigated the effect of resistance exercise training on strength and the protein expression or phosphorylation of factors important for upregulating (MyoD, myogenin, and IGF-I-R) and downregulating (activin receptor IIB (actRIIB) and smad2) skeletal muscle mass and mitochondrial markers in elderly compared to young individuals. In addition, we aimed at elucidating whether subsequent deconditioning affected these parameters in young and elderly similarly.

## 2. Materials and Methods

### 2.1. Subjects

Young and elderly volunteers were recruited with the criteria that for the elderly group age should be ≥74 years old and for the younger group age <30 years old. Exclusion criteria were non-sedentary status, illness requiring medical treatment other than treatment for hypertension and antithrombotic treatment, severe back or musculoskeletal pain, rheumatologic or neurological disorders, traumatic musculoskeletal and/or joint injuries, smoking, cardiovascular disease, attendance rate below 80% of total exercise sessions, additional exercise during the exercise phase, or failure to comply with instructions of inactivity during the deconditioning phase. Sedentary was defined as performing a maximum of three kilometers of cycling for transportation a day.

Twenty-two healthy, sedentary participants participated in the study; four were excluded due to noncompliance. Nine elderly, healthy persons—five men and four women (82 ± 7 year old)—fulfilled the inclusion criteria, and completed the resistance exercise training and deconditioning interventions. Data were compared to those found in nine young, healthy persons, also five men and four women (23 ± 3 yrs. of age). All participants completed a detailed medical history and had a normal neurological examination before entering the study. Demographic data of participants are shown in Table 1.

The study was approved by the Health Research Ethics Committee of the Capital Region of Copenhagen (No. KF-293615) and complied with the guidelines set out in the Declaration of Helsinki. The subjects were all informed about the nature and risks of the study and gave written consent to participate before inclusion.

### 2.2. Study Design

**Figure 1:**
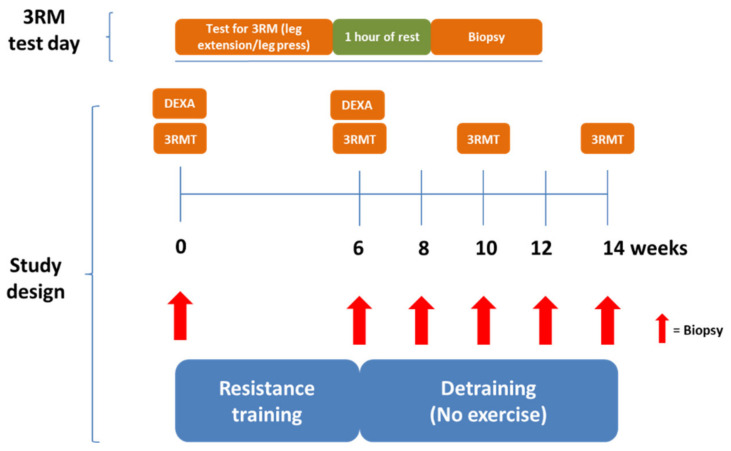
Study design overview. Eighteen participants completed a six-week resistance exercise training intervention followed by eight weeks of deconditioning (detraining—no exercise). Muscle strength was evaluated by a three-repetition max test before and after resistance exercise training. Skeletal muscle biopsies were taken from the vastus lateralis muscle on each test day. Furthermore, a biopsy was taken after two, four, and six weeks of deconditioning.

Nine young and 9 elderly participants completed a six-week resistance exercise training intervention of the lower body (two-legged knee extension) followed by eight weeks of deconditioning (Figure 1). Muscle strength was evaluated by a three-repetition max test before and after resistance exercise training. Skeletal muscle biopsies were taken from the vastus lateralis muscle before and after resistance exercise training and after two, four, six, and eight weeks of deconditioning for measurement of myogenic regulatory factors and mitochondrial markers. DEXA scanning of body composition was performed before and after resistance training.

### 2.3. DEXA Scanning

A whole-body Dual-Energy X-ray Absorptiometry (DEXA) scan (GE Medical Systems, Lunar, Prodigy, Chicago, IL, USA) was performed prior to the intervention and after the resistance exercise training intervention. The images were analyzed using enCORE™2004 Software (v.8.5) (GE Medical Systems). Reliability of this DEXA scanning was recently described [39].

### 2.4. Exercise Equipment and Protocol

Testing and exercise training intervention were carried out in a two-legged knee extension resistance exercise model using standard strength exercise equipment machines (Nordic Gym, Technogym, Cesena, Italy). Furthermore, to compensate for muscle imbalances during the selective exercise of the quadriceps, the exercise decline leg press (Nordic Gym) was incorporated into the exercise regimen.

### 2.5. Strength Testing

Strength testing was performed using a three-repetition maximum (RM) test-protocol. Before initial testing, participants were familiarized with the equipment and test protocol on a separate occasion to reduce the impact that skill learning has on strength performance. The estimated measure of bilateral knee extension muscle strength was recently found to be applicable for monitoring adaptations promoted by physical exercise for older adults with and without sarcopenia [40].

Three repetition maximum test (3RMT): Prior to the 3RMT, participants warmed up using five minutes of low intensity (60–80 watt and 30–50 watt for young and elderly, respectively) cycling ergometer exercise. Afterwards, participants were instructed to execute four repetitions in each attempt. Full range of motion (ROM) for three consecutive repetitions and failure to complete a 4th repetition across a full-ROM was set as a criterion for a successful 3RM estimate. Participants rested 3 min after warm-up, and 1.5 min between all other attempts. After 10 min of rest, the validity of the estimate was evaluated by trying to outperform the current 3RM estimate. 1RM was calculated using Brzycki’s formula [41]. 3RMT was measured before and after the resistance training intervention (Figure 1).

### 2.6. Resistance Exercise Training and Deconditioning Interventions

The resistance exercise training intervention lasted for six weeks and consisted of 16 supervised resistance exercise sessions. Resistance exercise followed a progressive protocol in weekly exercise sessions from two to three sessions per week after the first two weeks of exercise. Sessions were divided into three sessions of different load carried on a two-legged knee extension and a decline leg press machine to voluntary failure, and each set was separated by three minutes of rest. The first session encompassed three sets of 10–12 repetitions with 10–12 RM load, followed by three sets of 6–8 repetitions with 6–8 RM load, ending with three sets of 4–6 repetitions with 4–6 RM load. Each session was separated by approximately 48 h of rest.

In addition to and following knee extension resistance exercise, participants exercised in the decline leg press with the same exercise protocol (e.g., three sets of 10–12 reps with 10–12 RM in both exercises in the same session). After six weeks of resistance exercise training, participants stopped the exercise program and returned to their habitual sedentary lifestyle and were instructed not to initiate any new form of exercise the following eight weeks. This was ensured by participants wearing accelerometers and weekly interviews of the participants.

### 2.7. Skeletal Muscle Biopsy

A skeletal muscle biopsy was performed in vastus lateralis right leg muscle before and after the six weeks resistance exercise training intervention, and after two, four, six, and eight weeks of deconditioning (post2w, post4w, post6w, and post8w) approximately one hour post the acute 3RMT on the experimental days. The biopsy was performed as previously described using a 5 mm percutaneous Bergström needle [42]. Needle entry was at least three centimeters away from the previous insertion to avoid scar tissue and interference with data due to post-biopsy edema, regeneration, and cellular infiltration. Muscle samples were immediately frozen in isopentane cooled by liquid nitrogen before storage at −80 °C for later analysis.

### 2.8. Western Blotting Analysis

Western blot analysis was performed as previously described [43]. Biopsies were sectioned on a cryostat at −20 °C and homogenized in ice-cold lysis buffer with protease and phosphatase inhibitors (10 mM Tris, pH 7.4, 0.1% Triton-X 100, 0.5% sodium deoxycholate, 0.07 U/mL aprotinin, 20 M leupeptin, 20 M pepstatin, 1 mM phenylmethanesulfonyl fluoride (PMSF), 1 mM EDTA, 1 mM EGTA, 1 mM DTT, 5 mM β-glycerophosphate, 1 mM sodium fluoride, 1.15 mM sodium molybdate, 2 mM sodium pyrophosphate decahydrate, 1 mM sodium orthovanadate, 4 mM sodium tartrate, 2 mM imidazole, 10 nM calyculin, and 5 mM cantharidin; Sigma-Aldrich, St. Louis, MO, USA) using a Bullet Blender bead-mill at 4 °C (Next Advance, Averill, NY, USA). The homogenate was directly centrifuged at 15,000× *g* for 5 min at 4 °C. The supernatant was immediately transferred to new Eppendorf tubes and added 4× sample buffer including beta mercapto-ethanol. Equal amounts of extracted muscle proteins (10 μL) were separated on 4–15% polyacrylamide gels (Bio-Rad, Hercules, CA, USA) at 200 V for 40–50 min along with molecular weight markers (Bio-Rad). Proteins were transferred to PVDF membranes at 2.5 A for 5 min using a Trans-Blot Turbo (Bio-Rad) and blocked in Bailey’s Irish cream (R. J. Bailey & Co, Dublin, Ireland) for 30 min and washed in TBS-T to remove excess Bailey’s (3 × 10 min). The study investigated the expression and/or phosphorylation of proteins involved in muscle development/regeneration (IGF-I-R, MyoD, myogenin) and negative regulators of muscle mass (actRIIB and smad2) as well as porin [44], a mitochondrial membrane protein, to assess any changes in mitochondrial content. Thus, to investigate MRFs, antibodies against MyoD (45 kDa; diluted 1:1000; host: mouse; Thermo Fisher Scientific, Waltham, MA, USA) and myogenin (F5D) (40/25 kDa; diluted 1:1000; host: mouse; Developmental Studies Hybridoma Bank (DSHB), University of Iowa, IA, USA) were used. Antibodies against phosphorylated insulin-like growth factor 1 receptor (p-IGF-IR beta Y1135/1136; 95 kDa; diluted 1:500; host: rabbit; Cell Signaling Technology, Danvers, MA, USA), activin IIB receptor (58 kDa; diluted 1:1000; host: rabbit; ab180185, Abcam, Cambridge, UK), and phospho-smad2/3 (pSer250; 58 kDa; diluted 1:1000; host rabbit; Cell Signaling Technology) were used to investigate if muscle growth regulation had changed. Antibodies against porin (30–33 kDa; diluted 1:50,000; host: mouse; Thermo Fisher Scientific) were used to investigate a marker of mitochondrial content.

Antibodies against glyceraldehyde-3-phosphate dehydrogenase (GAPDH) were used at 1:5000 (ab22555; Abcam, Cambridge, UK) as loading control. Secondary goat anti-rabbit and goat anti-mouse antibodies coupled with horseradish peroxidase at concentration 1:10,000 were used to detect primary antibodies (DAKO, Glostrup, Denmark). Immunoreactive bands were detected by chemiluminescence using Clarity Max, (BioRad), quantified using a GBox XT16 darkroom, and GeneTools software was used to measure the intensities of immunoreactive bands (Syngene, Cambridge, UK). Immunoreactive band intensities were normalized to the intensity of the GAPDH bands for each subject to correct for differences in total muscle protein loaded on the gel.

### 2.9. Muscle Histology and Immunohistochemistry

Cryosections (10 µm) were cut from biopsies mounted in Tissue-Tek (Sakura Finetek Europe B.V., AJ Alphen aan den Rijn Netherlands), mounted on glass slides, and stored at −20 °C until stained. To assess myosin heavy chain (MHC) muscle fiber type distribution, sections were stained with MHC antibody clone BA-D5 (DSHB) for MHC type I, and a secondary goat anti-mouse antibody was used (GAM IgG2b Alexa Fluor 594, Thermo Fisher Scientific). For MHC type II assessment, sections were stained with MHC antibody clone A4.74 (DSHB), and a secondary goat anti-mouse antibody (GAM IgG1 Alexa Fluor 488) was used. All sections were observed at room temperature using a Nikon 20× Plan Apo VC N/A 0.75 mounted on a Nikon Ti-E epifluorescence microscope (Nikon Instruments, Melville, NY, USA). Images of the entire sections were acquired at 20× with a 5-Mpixel Andor Neo camera for fluorescence imaging (Andor, Belfast, Northern Ireland), using NIS-Elements Advanced Research (AR) software (Nikon Instruments) and merged in software.

### 2.10. Mitochondrial Citrate Synthase Enzyme Activity

Citrate synthase enzyme activity was investigated in muscle biopsies pre-exercise, post-exercise, and after 8 weeks of deconditioning. In short, skeletal muscle tissue (~200 mg) was sectioned on a cryostat (Microm HM550, Thermo Fisher, by, stat) at −20 °C and homogenized in ice-cold CelLytic MT (Mammalian tissue lysis/extraction reagent) containing protease inhibitor cocktail. The tissue was homogenized using a Bullet Blender bead-mill at 4 °C (Next Advance, Averill, NY, USA). The homogenate was directly centrifuged at 15,000× *g* for 5 min at 4 °C. The supernatant was transferred to new Eppendorf tubes and used for subsequent analysis. The assay was carried out according to the manufacturer’s instructions (#CS0720, Sigma-Aldrich). Briefly, the assay solutions were heated at 25 °C. A master mix consisting of 1× assay buffer, 30 mM Acetyl-CoA solution, and 10 mM DTNB solution were mixed and added in the lysate to perform triple measurements per sample. Citrate synthase was measured by reading absorbance at 412 nm every 10th second for 1.5 min, and thereafter adding 10 mM OAA solution and then remeasured again every 10 s for 1.5 min at 412 nm.

### 2.11. Statistical Analysis

**Figure 2:**
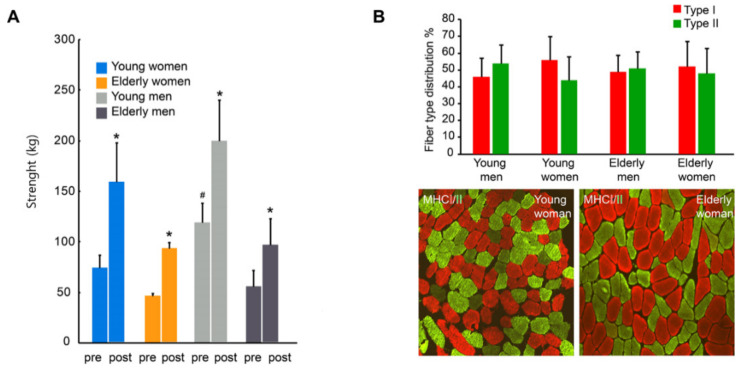
Muscle strength and muscle fiber type composition. (**A**) Quadriceps muscle strength measured in a three repetition maximum test before and after six weeks of resistance exercise training in elderly and young men and women. (**B**) Pre-intervention muscle fiber type distribution shown as bar graph and by representative cross-sectional images of m. vastus lateralis in a younger and older woman showing fiber type distribution of myosin heavy chain (MHC) type I (red) and type II (green). * *p* < 0.05, post-exercise vs. pre-exercise within the same group. # *p* < 0.05, pre-exercise, young men vs. young women. *n* = 5 elderly men, *n* = 4 elderly women, *n* = 5 young men, and *n* = 4 young women. All data are presented as means ± SD.

**Figure 3:**
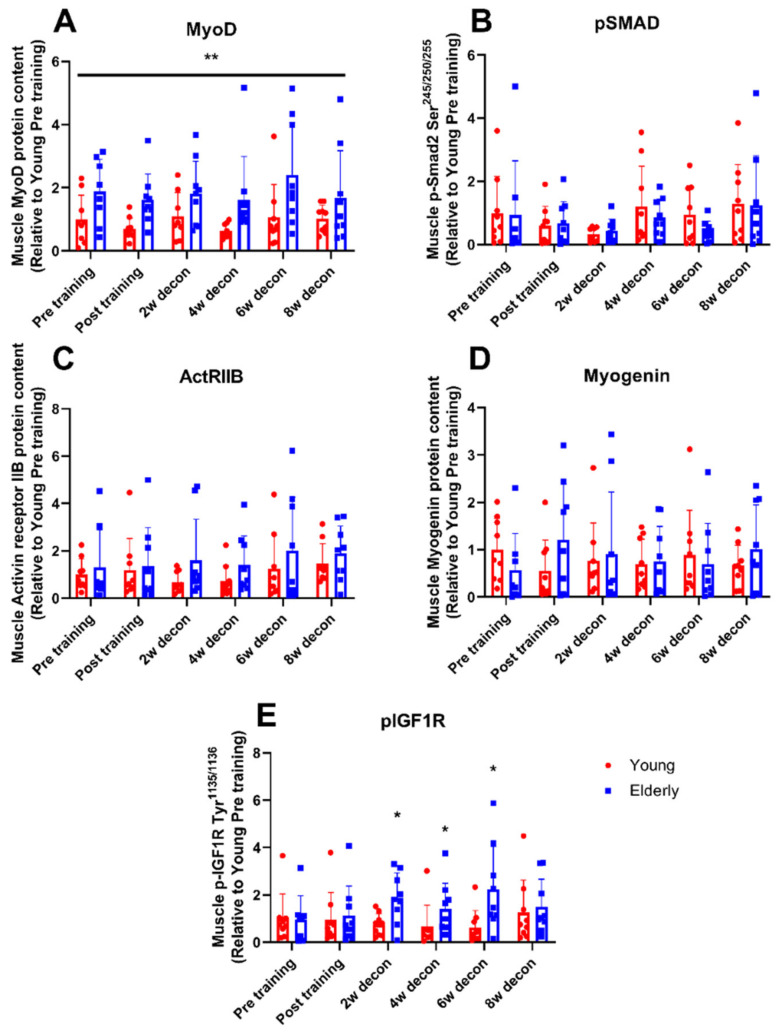
Muscle regulatory factors. Muscle regulatory factors measured in skeletal muscle pre and post six weeks of resistance exercise training and during 2, 4, 6, and 8 weeks into a subsequent deconditioning period in young (red bars) and elderly (blue bars) individuals. (**A**) Protein expression of activin receptor IIB, (**B**). Phosphorylation level of smad2 at Ser245/250/255. (**C**) Protein expression of MyoD. (**D**) Protein expression of myogenin. (**E**) Phosphorylation level of IGF-I-R at Tyr1135 /1136. Measurements have been performed as single determinations by Western blotting. Representative Western blots are shown in Supplementary Figure S1. *n* = 9 in young group and *n* = 9 in the elderly group. Values are arbitrary units (means ± SD) and expressed relative to young group pre training. * *p* < 0.05, young vs. elderly group, ** *p* < 0.01, main effect of age independently of training and deconditioning. ANOVA F-values (F_time_/F_age_/F_time×age_): (**A**) 1.08/11.62/0.32; (**B**) 0.76/0.58/0.49; (**C**) 0.77/2.97/0.23; (**D**) 0.09/0.15/1.20; (**E**) 0.54/8.62/1.41.

All statistical analyses were carried out using Excel 2010 (Microsoft^®^, Redmond, WA, USA) and GraphPad PRISM 8 (GraphPad, San Diego, CA, USA). A Shapiro–Wilkinson test was performed to test for normal distribution of data. Baseline subject characteristics were evaluated with unpaired *t*-tests between young and elderly groups and by a repeated measures two-way ANOVA for DEXA data before and after training (Table 1).The differences among groups were analyzed by a three-way repeated measures ANOVA in Figure 2A and a two-way repeated measures ANOVA in Figure 2B, Figure 3, and Table 2, followed by Tukey’s multiple comparison tests when ANOVA revealed significant interactions. Prior to this, an additional two-way ANOVA was performed to ensure no gender differences prior to pooling data for male and females.). Correlation analyses were performed with the Pearson’s product-moment correlation coefficient. All data are presented as means ± standard deviation (SD). Differences were considered to be statistically significant when *p* < 0.05.

## 3. Results

### 3.1. Anthropometry

Total body weight, body mass index (BMI), body fat %, and lean body mass % were similar between the young and the elderly group (Table 1). Six weeks of resistance exercise training did not lead to changes in whole body fat %, lean body mass %, or lean leg mass, as a read out for muscle mass, in the trained legs in neither the young nor the elderly individuals.

### 3.2. Muscle Strength

Six weeks of resistance exercise training increased quadriceps muscle strength in the elderly group by 78% (53.4 ± 14.3 kg (pre-exercise) vs. 95.0 ± 22.0 kg (post-exercise); *p* < 0.05), which was similar to that found in the young healthy persons (83.5%; 97.1 ± 27.5 kg (pre-exercise) vs. 178.2 ± 44.2 kg (post-exercise); *p* < 0.05) (Figure 2A). Pre-exercise, the elderly men did not have a significantly greater muscle strength compared to elderly women (56.2 ± 16.1 kg vs. 46.5 ± 2.5 kg), whereas a gender difference in muscle strength was observed in the younger group, in which the younger men had a 59% greater muscle strength at pre-training compared to younger women (119.2 ± 19.5 kg vs. 75 ± 12.6 kg; *p* < 0.05) (Figure 2A). No significant differences in strength between genders were observed post-exercise within the elderly or young group (Figure 2A).

### 3.3. Muscle Fiber Type Composition

No pre-exercise fiber type differences were seen between gender, young, and elderly participants (Figure 2B).

### 3.4. Myogenic Regulatory Factors

Six weeks of resistance exercise training and 8 weeks of deconditioning did not change the protein expression of MyoD, actRIIB, and myogenin and phosphorylation of Smad2 and IGF-I- receptor in the elderly and the young group (Figure 3A–E).

MyoD protein expression were overall higher (*p* < 0.01) in muscles of elderly compared with young individuals (Figure 3A).

No differences in total protein expression of actRIIB and myogenin and phosphorylation of Smad2 and IGF-I-R were observed pre-training or after six weeks following resistance training in young versus elderly participants (Figure 3B–E). IGF-I receptor phosphorylation at Tyr1135 /1136 was lower in the younger group of healthy persons compared to the elderly group two weeks post-exercise (post2w) (0.7 ± 0.5 vs. 1.9 ± 1.1, *p* < 0.05), four weeks post-exercise (post4w) (0.6 ± 0.9 vs. 1.4 ± 1.1, *p* < 0.05) and six weeks post-exercise (post6w) (0.6 ± 0.7 vs. 2.6 ± 2.7, *p* < 0.05) (Figure 3E). There was no difference between genders at all time points in both groups in all protein and phosphorylation levels investigated, why these data were pooled in the data shown.

### 3.5. Mitochondrial Markers: Porin and Citrate Synthase

The protein expression of porin did not change with six weeks of resistance exercise training and remained unchanged after deconditioning in both the young and the elderly and was not significantly different between the groups (Table 2).

The maximal muscle enzyme activity of citrate synthase in the elderly was not significantly different from that found in the young participants (Table 2). Maximal muscle enzyme activity of citrate synthase in the elderly group remained unchanged with resistance exercise training and subsequent deconditioning, which was similar to that found in the young group (Table 2). There was no difference in the citrate synthase activity among genders in the elderly and young groups, which is why these data were pooled (Table 2).

### 3.6. Correlation between Myogenic and Mitochondrial Factors and Muscle Strength

There was no association between any of the studied MRFs (MyoD, myogenin, actRIIB protein expression, and IGF-I-R and smad2 phosphorylation) or mitochondrial factors (citrate synthase activity and porin protein expression) and absolute or relative change in muscle strength after six weeks of resistance exercise training in the elderly or the young group.

## 4. Discussion

Resistance exercise training and deconditioning offer a unique opportunity to investigate differences in myogenic regulatory factors that may be crucial in the age-related loss of muscle mass and strength. The aim of this study was to investigate regulation of myogenic factors prior and in response to resistance exercise training and deconditioning in elderly (74–92 years of age) versus young, healthy, gender-matched individuals. The primary findings were (1) six weeks of intensive resistance exercise training induced the same increase in muscle strength in young and older individuals, (2) the key myogenic regulating factors (MyoD, myogenin, and IGF-I-R) were similar in muscles of young and elderly individuals and not differently regulated by six weeks of resistance exercise training or subsequent eight weeks of deconditioning, and (3) no change was found in markers of oxidative capacity and mitochondrial content after six weeks of resistance exercise training in either elderly or young healthy persons.

Age-related loss of muscle strength is inevitable and cannot be explained by age-related decreased physical activity level alone [45]. It has been suggested that elderly persons have a blunted muscle hypertrophy response to resistance exercise training [17,18,19]. Thus, it could be that differences in muscle mass and function between young and elderly are driven by age-related changes in the ability to gain and/or maintain muscle strength with age. However, the present study showed that six weeks of intensive resistance exercise training resulted in the same increase in quadriceps muscle strength in elderly compared to that found in young, gender-matched, sedentary individuals. Therefore, skeletal muscle of elderly has the same capacity to increase strength in response to resistance training as in young healthy individuals. This finding is important in a translational perspective, emphasizing that resistance exercise training benefits elderly as much as in young persons and therefore seems to be a valid tool to combat loss of muscle function and strength in aging.

Myogenic regulatory factors promote and regulate the expression of muscle-specific genes after muscle injury or strenuous resistance exercise leading to muscle hypertrophy [46]. It has been hypothesized that the inevitably age-related muscle strength loss may relate to downregulation of MRFs, and thus skeletal muscle atrophy, which we were unable to corroborate. With the same levels of MRFs in elderly compared to young persons in the present study, our findings contrast the majority of previous studies measuring on mRNA levels. Previous studies found elevated levels of MyoD, myogenin, and IGF-I-R mRNA in elderly both at rest [17,31,32,33,34] and in response to exercise training compared to younger individuals [17,47,48,49,50]. This suggests that the increase in MRF mRNA levels represented a continuous compensatory mechanism to preserve muscle protein and mass with aging [17]. The present study is the first to investigate protein levels of MRFs, obviating the issues with changes in mRNA levels that may not translate into similar changes in protein expression due to post-translational regulation [51]. The present study suggests that the myogenic program is intact in the elderly, as the levels of MyoD, myogenin, and IGF-I-R are activated to the same extent as in younger skeletal muscle. An overall higher level of MyoD protein expression in the elderly compared with the young muscle, and a higher IGF-I-R phosphorylation 2, 4, and 6 weeks into the deconditioning period in the elderly compared with young individuals support that compensatory mechanisms in MRF regulation contribute to preserve muscle protein and mass in aging. Importantly, our study underscores that protein and phosphorylation levels should be measured in favor of mRNA. Our finding further suggests that an intact anabolic muscle response seems able to compensate in part for the loss of muscle.

As the expression of MRFs are time-dependent in relation to external and internal stimuli, it could be hypothesized that lack of differences in MRFs in the present versus other studies (measuring mRNA levels though) investigating this could be related to the timing of the muscle biopsy sampling. In the present study, the muscle biopsy intervention was taken one hour post-exercise. As the expression of the proteins that was investigated in the present study is expected to be stable and not affected by an acute bout of exercise, timing of muscle sample seems not have an impact on the data presented in the present study. This is supported by findings in post-exercise muscle biopsy intervention (3 h post-exercise), in which no increase was found in key myogenic factors (MyoD, myf-6), except for myogenin [52], underscoring that timing of muscle biopsy sampling likely did not impact the protein levels of MRFs in the present study.

Muscle growth is tightly regulated through the myostatin, actRIIB, and smad2 pathway [53]. In response to muscle disuse, this and other pathways mediate a decrease in muscle mass [54]. In line with this, studies have shown that inhibition of myostatin-actRIIB-smad pathway leads to skeletal muscle hypertrophy in mice [55]. Studies by Hulmi et al. [35,36] have indicated that factors downregulating muscle mass in aged muscle could be attenuated in response to resistance exercise, as the mRNA level of actRIIB was downregulated after 21 weeks of resistance exercise in elderly (62.3 ± 6.3 years of age). The findings from that study indicate that proteins responsible for negative muscle mass regulation, e.g., actRIIB and smad2, could be upregulated in inactive aged muscle, resulting in decreased muscle mass and thus strength. However, our results did not support that finding. Instead, our data demonstrate that skeletal muscles of elderly have the same dynamics of MRF-mediated hypo- and hypertrophy, indicating that resistance exercise and deconditioning regulatory effects on skeletal muscle anabolism is the same irrespective of age.

Citrate synthase is a key mitochondrial matrix enzyme and a strong indicator of oxidative capacity in skeletal muscle [56,57,58,59] and maximal citrate synthase activity was also found to strongly correlate with mitochondrial volume measured by electron microscopy in skeletal muscles of healthy, young men [58]. Porin (also known as voltage dependent anion channel, VDAC1) is a pore-forming protein localized in the outer membrane of mitochondria, and is used as a marker of mitochondrial content [60,61]. Alterations in mitochondrial function and content has been proposed to be a factor underlying sarcopenia and muscle atrophy [62,63]. In order to investigate whether age-related decline in muscle strength was accompanied by muscle mitochondrial impairments in response to resistance training, muscle citrate synthase activity, and porin protein expression were measured before and after six weeks of resistance exercise training and eight weeks of deconditioning. Data showed that there was no change in oxidative capacity and indices of mitochondrial content after six weeks of resistance exercise training in either elderly or young healthy individuals, indicating that the gain in muscle strength was not associated with any changes in mitochondrial content.

It has been hypothesized that change in age-related muscle mass is driven by loss of muscle fiber type II number with age. However, findings regarding age-related changes in muscle fiber type II number have been ambiguous [64,65,66,67]. In the present study, the elderly individuals were older than those previously studied (+74 years old), and despite an age difference of 60 years between the young and elderly participants, there was no difference in number of type I and II fibers between elderly and younger persons. In the present study, we did not measure fiber type composition after training and deconditioning. Fiber type composition is in most studies not changed with exercise training [68] especially not within 6 weeks of resistance training. However, we cannot exclude that fiber type composition was mildly affected by exercise training in the present study. Furthermore, it was not within the scope of the present investigation to evaluate fiber size changes with resistance exercise training and deconditioning and it remains to be established, whether muscle fiber sizes are affected by 6 weeks of resistance training and subsequent 8 weeks of deconditioning in skeletal muscle of young and elderly.

## 5. Conclusions

Despite the fact that aging is associated with substantial loss of muscle mass resulting in a net loss of muscle strength and function, our study showed that elderly (aged 74+ years old) are remarkably capable of gaining muscle strength compared to younger participants in response to resistance exercise training. This underlines the applicability of resistance exercise training as an important instrument to diminish age-related loss of muscle function. Interestingly, the preserved ability to gain muscle strength with resistance exercise training was associated with a similar interaction between myogenic factors for up- and downregulation of skeletal muscle in elderly and young individuals. Thus, our data suggests that the entire myogenic regulatory program is not impaired in aged relative to younger skeletal muscle.

## Figures and Tables

**Table 1:** General demographic data.

	Young Group		Elderly Group	
Age, years	22.4 ± 2.2		82.3 ± 6.8 ***	
Height, cm	174.8 ± 10.4		167.5 ± 8.6	
Weight, kg	70.9 ± 15.1		64.7 ± 9.7	
BMI, kg/m^2^	22.9 ± 2.4		23.5 ± 2.9	
	Pre training	Post training	Pre training	Post training
Body fat (%)	26.2 ± 8.6	26.3 ± 9.5	27.9 ± 4.8	27.1 ± 5.8
LBM (%)	74.5 ± 8.9	74.5 ± 8.8	73.9 ± 6.6	73.7 ± 6.9
LLM (kg)	16.7 ± 4.2	17.0 ± 4.2	14.0 ± 2.9	14.2 ± 2.4

BMI, Body Mass Index; LBM, Lean Body Mass, LLM, lean leg mass. Age, height, weight, and BMI are presented at baseline pre intervention. Body fat, LBM, and LLM was measured before and after six weeks of resistance exercise training in elderly and young men and women. Data are shown as means ± SD. *n* = 9 in both groups. *** Significantly different (*p* < 0.001) from young group.

**Table 2:** Mitochondrial markers.

	Young Group			Elderly Group		
	Pre Training	Post Training	Post Deconditioning	Pre Training	Post Training	Post Deconditioning
Porin protein content relative to young pre training (AU)	1.00 ± 0.4	0.98 ± 1.1	1.60 ± 1.3	1.19 ± 1.2	1.53 ± 1.3	2.40 ± 1.9
CS activity, µmol/min/mg w.w.	1.7 ± 1.0	2.2 ± 1.3	2.0 ± 0.6	1.0 ± 0.6	1.7 ± 1.9	1.4 ± 0.9

Porin protein content and maximal muscle citrate synthase (CS) activity measured pre-training, following six weeks of resistance exercise training (post training), and after eight weeks of subsequent deconditioning (post decondition). Porin protein content is expressed relative to young pre training. Data are shown as means ± SD. *n* = 9 in both groups.

## Supplementary Materials

The following are available online at https://www.mdpi.com/2077-0383/9/7/2188/s1, Figure S1: Representative Western blots.
