# Supplementary figures and images for: Preserved Capacity for Adaptations in Strength and Muscle Regulatory Factors in Elderly in Response to Resistance Exercise Training and Deconditioning
Source: J Clin Med. 2020 Jul 10;9(7):2188. doi: 10.3390/jcm9072188 (PMC7408999; doi:10.3390/jcm9072188)

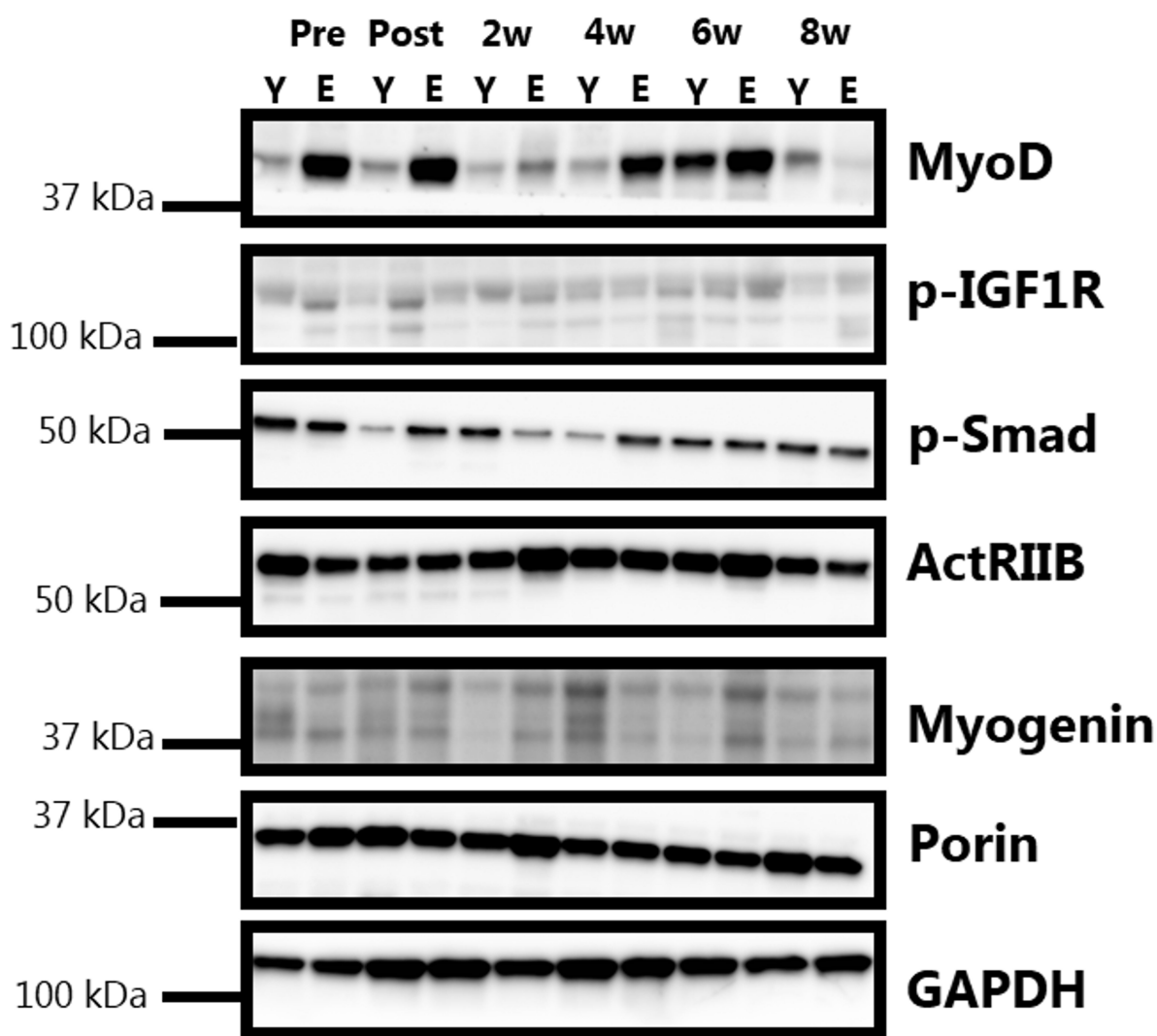

**Supplementary figure 1.** Representative Western blots.

Supplement: Supplementary file 1 [file jcm-09-02188-s001.pdf]
